# New Perspectives in the Etiology of Chronic Rhinosinusitis—Haller Cells

**DOI:** 10.3390/medicina60111867

**Published:** 2024-11-14

**Authors:** Alin Horatiu Nedelcu, Razvan Tudor Tepordei, Ancuta Lupu, Vasile Valeriu Lupu, Marius Constantin Moraru, Simona Alice Partene Vicoleanu, Gabriel Statescu, Cosmin Gabriel Popa, Manuela Ursaru, Cristina Claudia Tarniceriu

**Affiliations:** 1 Department of Morpho-Functional Science I, Discipline of Anatomy, “Grigore T. Popa” University of Medicine and Pharmacy, 16 Universitatii Street, 700115 Iasi, Romania; alin.nedelcu@umfiasi.ro (A.H.N.); razvan.tepordei@umfiasi.ro (R.T.T.); marius.moraru@umfiasi.ro (M.C.M.); partene.vicoleanu@umfiasi.ro (S.A.P.V.); gabriel.statescu@umfiasi.ro (G.S.); cosmin-gabriel.popa@umfiasi.ro (C.G.P.); claudia.tarniceriu@umfiasi.ro (C.C.T.); 2 Radiology Clinic, Recovery Hospital, 700661 Iasi, Romania; 3 Department of Mother and Child, “Grigore T. Popa” University of Medicine and Pharmacy, 16 Universitatii Street, 700115 Iasi, Romania; 4 Department of Surgical Sciences I, “Grigore T. Popa” University of Medicine and Pharmacy, 16 Universitatii Street, 700115 Iasi, Romania; manuela.ursaru@umfiasi.ro; 5 Radiology Clinic, “Sf Spiridon” County Clinical Emergency Hospital Iasi, 700661 Iasi, Romania

**Keywords:** Haller cells, chronic rhinosinusitis, maxillary sinus, maxillary infundibulum, ethmoid air cells

## Abstract

*Background and Objectives*: Haller Cells (HCs) represent the abnormal migration of ethmoid cells that are located below the ethmoid bulla at the level of the upper wall of the maxillary sinus. Through their placement, the cells can exert a mass effect on the infundibulum of the maxillary sinus. The aim of our study is to investigate the prevalence of Haller cells in the Romanian population and to evaluate the relationship between this anatomical variation and chronic rhinosinusitis. Secondly, we want to morphometrically evaluate the impact of Haller cells’ presence in the drainage paths of the maxillary sinuses. *Materials and Methods*: We conducted a randomised retrospective study that included 255 consecutive multi-detector computed tomography (MDCT) scans of the head. To carry out a comparative evaluation of the association of Haller cells with chronic rhinosinusitis, we divided the patients into two subgroups, a seasonal-based sample, between November 2022 and January 2023, and June 2023 and August 2023, respectively. We report the mean ± standard deviation for the continuous variables. To compare the results, we used the following statistical tests: a chi-squared test and a paired Student’s *t*-test (one-tail). *Results*: Our study identified a high prevalence of Haller cells, namely in 128 out of 255 patients (50.2%). There were no statistically significant associations between the presence of HCs and the age and the gender of the sample, respectively. The unilocular morphotype predominates in unilateral shapes and in bilateral shapes as well (*p* = 0.002). Our study identified the correlation between the Haller cells and the chronic rhinosinusitis in both research samples: “Winter group” and “Summer group” (*p* = 0.0002 and *p* = 0.0001, respectively). *Conclusions:* It was determined that for 40 out of 42 patients, the presence of HCs changes the morphometric pattern of the infundibular maxillary sinus.

## 1. Introduction

The pneumatised ethmoid-sinus complex is an anatomical structure that belongs to the viscerocranium. Its functional importance is given by the fact that it creates the limitation between the airways, the orbit, and the dental alveoli. As such, a pathological process occurring in any of these structures can directly extend itself to the others [1].

Haller cells (HCs) or “Cellula Halleri” have been initially described by the Swiss anatomist Albrecht von Haller in 1743, naming them *Cellulae orbitariae*. These elongated and narrow cells are located between the planum bone and the superior maxillary bone. At the same time, Haller highlighted the fact that the cells are not necessarily unique, as multilocular shapes can exist. The first researcher that determined a nominal connection between the infraorbital cells and Albrecht von Haller was the popular Austrian anatomist Joseph Hyrtl, who used the term “Cellulae orbitariae Halleri” in 1846. Other two important anatomists of the 19th century, Jacob Henle and Emil Zuckerkandl, highlighted the existence of these cells in 1871 and in 1882, respectively. During the dissection process, Zuckerkandl observed the mass effect of HCs on the infundibulum of the maxillary sinus. Simultaneously, he mentioned for the first time the causal relationship between the presence of these cells and the infectious rhinosinusitis pathology [2].

Similarly to other developmental anomalies of the pneumatised structures of the face, such as concha bullosa, Onodi cells, or Agger nasi cells, Haller cells associate the abnormal migration of the ethmoid cells with their disposal at the ceiling level of the maxillary sinuses. The origin of the cells, which migrate in order to form HCs, can be in the anterior and posterior ethmoid cells, a structure also found in the concha bullosa [2,3,4,5]. The placement between the inferior wall of the orbit and the infundibular maxillary sinus determines morphological and morphometrical changes in the communication system between the sinus cavity and the middle meatus. The dimensional changes in the infundibulum are directly dependent on these cells’ positioning and especially on their dimensional characteristics. In addition, through the mass effect created on the infundibulum, the Haller cells influence the mucociliary drainage and represent a favourable factor in the recurrent infectious rhinosinusitis pathology [2,6,7,8]. In addition to sinus infectious pathology, the presence of Haller cells is often associated with headaches and mucocele. In the case of expansive forms, local compression, nasal obstruction, and rhinogenic orofacial pain may occur [9]. Başer et al. [10] identified a high frequency of Haller cells (7 out of 54) in patients diagnosed with antrochoanal polyposis and concluded that variations in rhinosinusal pneumatization through airflow distortions may be an etiological factor.

The methods used to diagnose rhinosinusitis pathology include the clinical exam, represented by the direct rhinoscopy, and fibroscopy, completed by the imagistic exploration. Conventional radiography offers information about the inflammation of the skull’s pneumatised cavity and of the associated regions, such as the spine. By setting acquisitions with fine sections (1 mm-section thickness or less) and tridimensional reconstructions, modern imagistic methods, such as multi-detector computed tomography (MDCT), offer detailed pictures of the section of interest. Hence, multiple anatomical variations are present at the level of the craniocerebral and the superior cervical regions. The only limitation is represented by a higher rate of irradiation compensated by the new generations of machines, which minimise this deficiency. Cone Beam Computed Tomography (CBCT) is another modern method that decreases the irradiation rate while offering useful pictures to determine the diagnosis of the sinus pathology. Magnetic Resonance Imaging (MRI) excludes the radiation X’s effects and offers highly accurate images, especially concerning the soft tissue; however, it involves increased costs [11,12,13,14,15].

The aim of our study is to investigate the prevalence of Haller cells in the Romanian population and to evaluate the relationship between this anatomical variation and chronic rhinosinusitis. Moreover, we want to morphometrically evaluate the impact of the presence of Haller cells on the drainage paths of the maxillary sinuses.

## 2. Materials and Methods

### 2.1. Study Design

We conducted a randomised retrospective study that included 255 consecutive head MDCT scans. To carry out a comparative evaluation of the association of Haller cells with chronic rhinosinusitis, we divided the patients into two subgroups, a seasonal-based sample, between November 2022 and January 2023, and June 2023 and August 2023, respectively. The two subgroups are similar in terms of time (three months each) but different in terms of season (summer and winter, respectively). The study was conducted at the Clinical Recovery Hospital of Iasi, and it was approved by the local Ethics Committee, approval no. 11/16.02.2024. Our research is based on clinical diagnostic protocols. All patients included in the study signed an informed consent in accordance with the Declaration of Helsinki.

### 2.2. Materials

Images were acquired using a Toshiba Aquilion 16 CT Scanner (Toshiba Romania Representative, Iasi, Romania) and were processed using the Radiant DICOM Viewer software 2023. A multi-slice helical scan with axial, coronal, and sagittal reconstructions was performed in the bone window at 0.6 mm. To avoid mistakes in identifying inclusion and exclusion criteria and to minimise acquisition bias, two experienced radiologists independently evaluated each dataset. In case of inconsistency, a third radiologist intervened, and the result was traded after everyone’s approval.

Haller cells are defined as an abnormal pneumatization of inferiorly migrated ethmoid cells from the ethmoid bulla. It is essential that these cells are located on the upper wall of the maxillary sinus, adjacent to the *lamina orbitalis* or *lamina papyracea*. Medially, the intersinusonasal wall and the uncinate process delimit them. Appendages of the ethmoid bulla, which are extended to the ceiling of the maxillary sinus, are not considered Haller cells.

The clinical diagnosis of chronic rhinosinusitis implies the extension of sinus symptoms for more than 12 weeks. In the absence of these criteria, being a retrospective study, we used imaging criteria, namely the presence of thickened mucosa in the sinus cavities greater than 4 mm associated with hyperostosis (sclerotic thickened bone).

### 2.3. Inclusion and Exclusion Criteria

All patients over 18 years of age without traumatic injuries of the neurocranium and viscerocranium were included in the study. Images showing various types of artefacts and images that did not completely capture the maxillary sinuses were not taken into account. We excluded the patients who underwent ENT or neurosurgical interventions.

### 2.4. Statistical Analysis

Excel 2016 software for Windows was used for the statistical analysis. We report the mean ± standard deviation for the continuous variables. To compare the results, we used the following statistical tests: a chi-squared test and a paired Student’s *t*-test (one-tail). Pearson’s chi-squared test was employed for comparisons between groups in categorical variables. *p*-values below 0.05 are considered statistically significant.

## 3. Results

### 3.1. Baseline Characteristics of Patients

Our retrospective study includes head CT scans of 255 patients, aged between 18 and 92 years old. The age series is homogeneous so that statistical tests can be applied: mean age is 60.40 years ± 18.37, median age is 65 years, skewness test *p* = −0.616 (Table 1).

In our cohort, men predominate (140 out of 255) with a percentage of 54.9% compared to women 45.1% (115 out of 255), without identifying a statistically significant difference (*p* = 0.10). According to age, a slightly advanced age can be identified in women (63.66 years ± 17.33) compared to men (57.73 years ± 18.83). The statistical analysis signifies the homogeneity of the studied batch. In conclusion, we can apply all comparative statistical tests.

### 3.2. Haller Cell Prevalence by Gender

Haller cells were identified in 128 out of 255 patients, representing 50.2%. Of these, 74 out of 140 patients (52.85%) were identified as males, while 54 out of 115 patients were female (45.09%). There were no statistically significant differences between the two genders, *p* = 0.348 (Table 2).

### 3.3. Location of Haller Cells According to Gender

**Figure 1:**
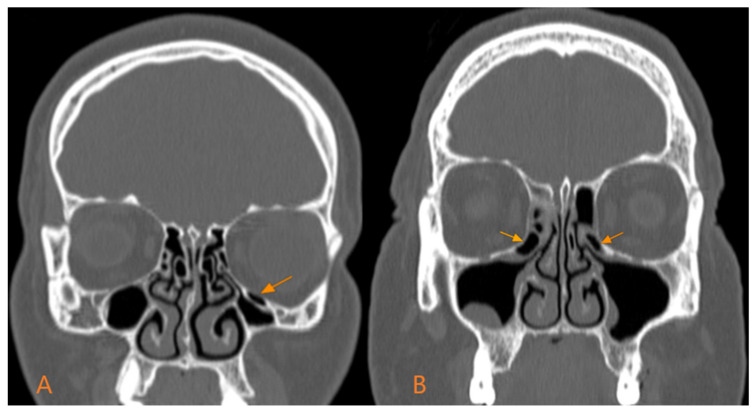
Types of Haller cells according to location: (**A**)—unilateral; (**B**)—bilateral.

We did not identify statistically significant differences regarding the unilateral or bilateral localisation depending on gender (*p* = 0.66) (Figure 1). In men, the bilateral type prevailed, with 65% (48 out of 74), while the unilateral type was in the minority, with 35% (26 out of 74). This pattern was also preserved in women, with 37 out of 54 (68.5%) presenting the bilateral type and only 17 out of 54 (31.5%) the unilateral type (Table 3).

### 3.4. Haller Cells: Morphologic Types According to Gender and Location

**Figure 2:**
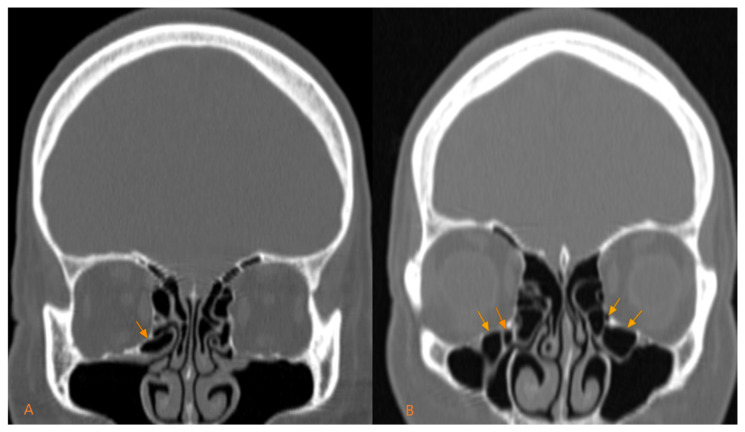
Morphological types of Haller cells: (**A**) unilocular; (**B**) multilocular.

Regarding the morphological type, the predominant type is the unilocular type, namely 66.19% (141 out of 213), compared to the multilocular type, which is 33.81% (72 out of 213) (Figure 2). Their prevalence by gender follows the same distribution, in men, the unilocular type is predominant, present in 78 out of 122 cases (63.93%), compared to the multilocular type, present in 44 out of 122 cases (36.06%). In women, the unilocular type represented 69.23% of cases (63 out of 91) compared to the multilocular type, which represented 30.76% of cases (28 out of 91). The statistical analysis did not show significant differences between the two genders for the morphological types, *p* = 0.418 (Table 4).

The unilocular morphological type is predominant in both unilateral (37 out of 43) and bilateral (104 out of 170) forms. The multilocular type is a minority both in bilateral locations (66 out of 170) and in unilateral locations (6 out of 43). Regarding the distribution of morphological forms according to location, a statistical significance is reached (*p* = 0.002), so that we can conclude that the unilocular type predominates (Table 5).

### 3.5. The Relation of Haller Cells and Chronic Rhinosinusitis

In order to evaluate the association between the presence of Haller cells and chronic rhinosinusitis, we divided our cohort group into two seasonal subgroups. We named the first group the “winter group” because it consists of 133 patients selected between November 2022 and January 2023. It is characterised by a mean age of 59.39 ± 19.82 and a higher proportion of men (75 out of 133) versus women (58 out of 133). We named the second subgroup the “summer group” being made up of 122 patients selected between June 2023 and August 2023 with a mean age of 61.50 ± 16.68. There is also a slight male predominance (65 out of 122) compared to women (57 out of 122). There were no statistical differences between the two subgroups in terms of age (*p* = 0.36) or gender distribution (*p* = 0.61). Within each subgroup, the two maxillary cavities were evaluated separately in terms of the presence or absence of Haller cells and whether or not they were associated with chronic rhinosinusitis. In total, there will be 266 maxillary cavities in the “winter group” and 244 maxillary cavities in the “summer group”.

In the “Winter group”, we identified 125 cavities with Haller cells, out of which 86 (68.80%) were associated with rhinosinusitis, while only 39 (31.20%) did not show CT signs of rhinosinusitis. Among the cavities where Haller cells were not identified, 66 out of 141 were associated with chronic rhinosinusitis, while 75 out of 144 had no lesions suggestive of chronic rhinosinusitis. The statistical analysis (chi-squared test) demonstrated a high significance (*p* = 0.0002), and thus we can conclude that in the “winter group” there is a correlation between the presence of Haller cells and the CT signs of chronic rhinosinusitis (Table 6).

A number of 122 patients and 244 cavities were investigated in the “Summer group”. In total, we identified 88 cavities with Haller cells, while the remaining 156 out of 244 were categorised as normal. In the presence of Haller cells, we identified 50 cases (56.81%) with specific CT signs for chronic rhinosinusitis and 38 out of 88 cases (43.18%) without such signs. Of the sinus cavities that do not show Haller cells (156 out of 244), 49 out of 156 (30.43%) have specific signs of chronic rhinosinusitis, while the majority, 107 out of 156 (68.58%), do not show such signs. The statistical analysis (chi-squared test) carried out suggests a high significance, namely *p* = 0.0001 (Table 7).

### 3.6. The Influence of Haller Cells on the Dimensional Characteristics of the Maxillary Infundibulum

As a further step, our study investigates how Haller cells dimensionally influence the ostiomeatal complex (OMC). The ostiomeatal unit consists of the maxillary ostium, infundibulum, and hiatus semilunaris. The infundibulum is the longest but also the narrowest segment of the OMC. We measured the infundibulum bilaterally at its narrowest point. We selected the patients identified with unilateral Haller cells, taking into account the relative symmetry of the skeleton of each individual. Thus, we obtained a batch of sinus cavities that are associated with Haller cells and a second batch that is not associated with Haller cells. Each selected patient has one sinus component (infundibulum) in each of the two groups. A total of 42 maxillary sinus units were included in each batch. One patient identified with hypoplasia of the left maxillary sinus was excluded from the study. The mean diameter of the maxillary infundibulum measured in association with Haller cells is 1.085 ± 0.403 mm. For sinus cavities that are not associated with Haller cells, the mean diameter of the maxillary infundibulum is 1.642 ± 0.682 mm (Table 8). In 2 out of 42 cases, the infundibulum is wider in association with Haller cells, compared to the opposite side of the infundibulum that is not associated with Haller cells.

Despite the fact that in the vast majority of cases (40 out of 42), the infundibulum of the maxillary sinus associated with Haller cells was smaller than the one on the opposite side (without Haller cells), the Paired Student *t*-Test One Tail did not demonstrate a significance (*p* > 0.05). The statistical result was definitely influenced by the small number of selected cases.

## 4. Discussion

Our study group, which included 255 patients, is balanced considering the gender distribution with no significant differences between males and females. According to the Haller cells’ prevalence in the study sample, not fewer than 128 patients (54.9%) out of the 255 patients, which met the inclusion and exclusion requirements, showed this type of anatomical variant. Our results are in line with the results obtained by Yousefi et al. [12], Azila et al. [16], and Alsowey et al. [17], with a prevalence of 57.3%, 55.6%, and 61.3%, respectively. These studies used MDCT as an exploratory technique.

In contrast to the studies accomplished using MDCT, a category that includes our study as well, the ones that used the CBCT highlighted a lower incidence of Haller cells. The lower resolution of the images acquired can represent a factor that influences the detection of some small shapes [12,14,18,19,20].

Giaccaglia et al. [15] identified HCs in only one patient out of those 61 patients included in his research, using CBCT. The same study identified decreased incidents related to the other anatomical variants of the ethmoid meatal, such as concha bullosa (7% compared with 53.77% in Nedelcu et al. [5]) or Onodi cells (23% compared with 55.8% in Alsowey et al. [17]). The decreased incidence can be attributed to the technique used and especially to the characteristics of the studied sample, which was represented by children aged 5 to 14 years old. In this case, the ostiomeatal complex is not yet built, with some loco-regional malformations developing later on.

Atsal et al. [21] identified a causal relationship between a deviated septum and the presence of the Haller cells. In his study, he proved that the incidence of HCs and of Onodi cells is higher for patients with a case of deviated septum of more than 15%. At the same time, the incidence of the other ostiomeatal malfunctions, such as concha bullosa or agger nasi, does not seem to be influenced by the deviation of the nasal septum.

From a gender breakdown perspective, our study did not prove significant statistical differences. In the case of males, 74 out of 140 patients (52.85%) showed Haller cells, while in the case of females, 54 out of 115 patients (46.95%) were identified with this anomaly. The obtained results are in line with the ones published by most authors [7,22,23]. We identified a singular study that mentions a higher frequency of HCs in females’ sample (female/male ratio 3:1) [19].

The predominant location was the bilateral one, at 66.40% (85 out of 128), in contrast to the unilateral placement, at 33.59% (43 out of 128) and uniformly distributed by gender. In our cohort, significant differences related to the morphological shape were not recorded as well; however, unilocular shapes predominated, at 66.19% (141 out of 213), in contrast to the multilocular shapes, at 33.80% (72 out of 213). Our results are not in line with those obtained by other researchers, such as Khojastepour et al. [7], who identified the unilateral shape in 36 out of 50 HC-positive patients and the bilateral shape in 14 out of 50 patients. Pekiner et al. [23] also described the unilateral shape in 47 out of 65 patients and the bilateral shape in 18 out of 65 patients. Our study’s advantage is represented by the high number of subjects included in the sample, but also by acquisitions with fine sections (0.6 mm).

Moreover, our study proved a higher incidence of the unilocular morphological type, in contrast with the multilocular one, in the case of a unilateral placement (37 vs. 6 out of 43) and a bilateral one as well (104 vs. 66 din 170), *p* = 0.002. To our knowledge, in the literature there is no comparative evaluation of the morphological types with respect to the localisation type.

The causal relationship between the presence of the Haller cells and the chronic rhinosinusitis raises numerous disputes. Some researchers, such as Khojastepour et al. [7], Ali et al. [20], or Kamdi et al. [24], obtained results with statistical significance regarding the association between HCs and rhinosinusitis, while others did not highlight this outcome [19,22]. The study led by us investigates the link between the presence of HCs and chronic rhinosinusitis, using an original protocol. The patients were divided into two groups according to two periods of 3 months. Each period corresponds to a season with a diametrically opposite incidence of acute infections from the ENT sphere. With the seasonal chosen sample, we aim to provide unbiased results related to acute rhinosinusitis. In the two study samples, every patient who met the inclusion requirements was selected. If the two groups proved to be similar from an age perspective, the gender, and the number of patients, the causal relationship between the presence of HCs and chronic rhinosinusitis was proven by the statistical tests for the “winter group” (*p* = 0.0002) and for the “summer group” (*p* = 0.0001). We can thus conclude that the presence of Haller cells is a favourable factor for the occurrence of chronic rhinosinusitis, regardless of the environmental factors and the seasonal distribution.

From our point of view, one of the major causes for the occurrence of the chronic rhinosinusitis pathology is represented by the impairment of mucociliary drainage. Heller cells can influence mucus elimination from the maxillary sinuses’ cavities by the mass effect exerted on the infundibulum. As such, we aimed to evaluate from a morphometric perspective the effect on the maxillary infundibulum through the presence of the Haller cells. In order to obtain a clear image of this impairment, we included in our study only the patients who were identified with unilateral shapes (42 patients). The studied sample is represented by the hemifacies that present HCs. The control group is represented by the hemifacies on which there was no indication of Haller cells. Taking into account the relative symmetry of the skull, we consider that the morphometric changes are more eloquent if the comparison is made on the same patients, included both in the studied sample and the control sample. Even though statistical significance was not achieved, our study identified that for 40 out of 42 patients, the diameter of the infundibulum was reduced compared to the incentive one (1.085 ± 0.403 mm vs. 1.642 ± 0.682 mm). Our study is in line with the one led by Basurrah et al. [25], concluding that the presence of Haller cells causes morphometric changes in the infundibulum.

The association of Haller cells with chronic rhinosinusitis implies a complex and multimodal treatment. The first therapeutic line is represented by conservative treatment consisting of the combination of intranasal corticosteroids and washing with saline solutions. Antihistaminic and mucolytic medication is added to this. Antibiotic treatment is required in bacterial superinfection confirmed after endoscopic sampling of secretions from the middle meatus [26,27].

Surgical treatment is required in case of failure of correctly conducted conservative treatment, chronic recurrent rhinosinusitis, the association of nasal polyposis, or the appearance of complications. The goal is to restore sinus drainage. The chosen method is functional endoscopic sinus surgery. In the presence of expansive Haller cells, their resection can also be attempted [26,27,28].

### 4.1. Research Frontiers

The placement of the Haller cells at the verge between the orbital floor and the maxillary sinus’ ceiling modifies the morphological loco-regional appearance. Through the mass effect exerted on the infundibulum, HCs can lead to the impairment of the mucociliary drainage, particularly evident in the case of voluminous and multilocular types.

### 4.2. Innovations and Breakthroughs

Our study’s strength emerges from the comparison analysis of the two samples with seasonal distribution. In this way, we assessed the association between Haller cells and rhinosinusitis, independently from environmental factors. Another advantage is the inclusion in the morphometric study of the patients with the unilateral presence of the Haller cells. Taking into consideration the relative symmetry of the viscerocranium, we assume that selecting the control group from the same patients as those belonging to the studied group is more trustworthy than selecting a random control group.

### 4.3. Limitations

Our study has some limitations. Firstly, the number of cases must be increased, especially considering the evaluation of the maxillary sinus’ infundibulum. Secondly, being a retrospective study, imaging diagnostic criteria for chronic sinusitis were used, and clinical criteria were elided. Thirdly, a detailed morphological and morphometrical description of the ostiomeatal complex is needed, an idea that would serve as a basis for future studies.

## 5. Conclusions

Haller cells represent a placement anomaly of ethmoid cells with a marked prevalence in the general population. Factors such as age and gender do not influence the incidence of this development anomaly. The unilocular type is predominant in both unilateral placement and bilateral placement. The causal relationship between Haller cells and chronic rhinosinusitis was proved in both studied samples: “Winter group” and “Summer group”.

## Figures and Tables

**Table 1:** Descriptive statistics of age.

Mean	60.40784314
Standard Error	1.150853939
Median	65
Mode	72
Standard Deviation	18.37766364
Sample Variance	337.7385209
Kurtosis	−0.38911299
Skewness	−0.616121178
Range	74
Minimum	18
Maximum	92
Sum	15404
Count	255
Confidence Level(95.0%)	2.266431373

**Table 2:** Haller cell prevalence by gender.

	Haller Cell	Non-Haller Cell	Sum	
Male	74	66	140	*p* = 0.348
Female	54	61	115	
Total	128	127	255	

**Table 3:** Haller cells: location according to gender.

	Unilateral	Bilateral	Sum	
Male	26	48	74	*p* = 0.665
Female	17	37	54	
Total	43	85	128	

**Table 4:** Haller cells: morphologic type according to gender.

	Unilocular	Multilocular	Sum	
Male	78	44	122	*p* = 0.418
Female	63	28	91	
Total	141	72	213	

**Table 5:** Haller cells: morphological type according to location.

	Unilocular	Multilocular	Sum	
Bilateral	104	66	170	*p* = 0.002
Unilateral	37	6	43	
Total	141	72	213	

**Table 6:** Seasonal association between Haller cells and rhinosinusitis—“Winter group”.

	Haller Cells	Non-Haller Cells	Sum	
Rhinosinusitis	86	66	152	*p* = 0.0002
Non-rhinosinusitis	39	75	114	
Total	125	141	266	

**Table 7:** Seasonal association between Haller cells and rhinosinusitis—“Summer group”.

	Haller Cells	Non-Haller Cells	Sum	
Rhinosinusitis	50	49	99	*p* = 0.0001
Non-rhinosinusitis	38	107	145	
Total	88	156	244	

**Table 8:** The influence of Haller cells on the dimensional characteristics of the maxillary infundibulum.

	Maxillary Ostium + Haller Cells	Maxillary Ostium + Non-Haller Cells
Patients	42	42
Mean	1.08547619	1.642142857
Standard Deviation	0.403800255	0.682161327
Median	1.035	1.53
Minimum (mm)	0.49	0.66
Maximum (mm)	2.07	3.62
Confidence Level (95.0%)	0.125833018	0.212576434

## Data Availability

All relevant data are contained within the manuscript. The raw data supporting the conclusions of this article will be made available by the authors on request.
